# Prise en charge des complications des fistules artério-veineuses pour hémodialyse chronique

**DOI:** 10.11604/pamj.2015.20.202.3617

**Published:** 2015-03-05

**Authors:** Hamid Jiber, Youssef Zrihni, Rachid Zaghloul, Rita Hajji, Othman Zizi, Abdellatif Bouarhroum

**Affiliations:** 1 Service de Chirurgie Vasculaire, CHU Hassan II, Fès, Maroc

**Keywords:** Fistules artério-veineuses, hémodialyse, complications, chirurgie, endovasculaire, arteriovenous fistulas, hemodialysis, complications, surgery, endovascular

## Abstract

La fistule artério-veineuse native est l'accès vasculaire de choix pour l'hémodialyse chronique en raison de sa longévité, son taux faible de complication et de mortalité par rapport aux pontages artério-veineux et aux cathéters. Cependant, il arrive assez souvent que l'on assiste à des complications qui sont dominées par la sténose et la thrombose. C'est une étude rétrospective des complications ayants survenues pour 31 fistules artério-veineuses pour hémodialyse chronique des 200 fistules réalisées chez 200 patients au sein du service de chirurgie vasculaire du CHU Hassan II de Fès sur une période de trois ans, étendue de Janvier 2007 à Décembre 2009. Ces complications ont été présentés par les thromboses dans 14 cas soit 45,15% de l'ensemble des complications, les sténoses dans 4 cas (12,90%,) les anévrismes dans 4 cas (12,90%), les complications ischémiques dans 3 cas (9,67%), l'infection dans 3 cas (9,67%), l'hémorragie dans 2 cas (6,45%) et l’ hyperdébit dans un seul cas soit 3,22%. On a pu conserver 22 fistules soit 70,96% par traitement chirurgical ou endovasculaire, on a confectionné une nouvelle fistule dans 8 cas soit 25,80%, et on a adressé une patiente (3,22%) pour pose d'un cathéter veineux tunnelisé permanent. Les complications des fistules artério-veineuses pour l'hémodialyse chronique sont la principale cause de morbidité chez les patients hémodialysés, il est donc important de s'impliquer lors de leur création, et de donner un maximum d'attention quand ils sont manipulés. Ceci suggère la mise en place d'un programme de surveillance de ces fistules en raison de l'impact des complications sur la morbi-mortalité du patient hémodialysé et sur le plan financier.

## Introduction

La fistule artério-veineuse (FAV) native est l'accès vasculaire de choix pour l'hémodialyse chronique en raison de sa longévité, son taux faible de complication et de mortalité par rapport aux pontages artério-veineux et aux cathéters. Cependant, il arrive assez souvent que l'on assiste à des complications qui sont dominées par la sténose et la thrombose [1].

## Méthodes

C’était une étude rétrospective des complications ayants survenues pour 31 fistules artério-veineuses réalisées chez 31 patients au sein du service de chirurgie vasculaire du CHU Hassan II de Fès sur une période de trois ans, étendue de Janvier 2007 à Décembre 2009. Ces complications ont été présentées par le (Tableau 1). Les thromboses: Dans notre série, la thrombose était la cause la plus fréquente de la perte de la FAV, elle a intéressé 14 FAV soit 45,16% de l'ensemble des complications. Nous avons considéré comme précoces, les thromboses survenues dans le 1^er^ mois ayant suivi la création de la FAV. Nous avons, ainsi défini, constaté 6 épisodes de thromboses précoces chez 6 patients, et 8 épisodes de thromboses tardives chez 7 patients. Le diagnostic de la thrombose était clinique, le fait important étant la disparition du thrill. Les sténoses: Quatre FAV se sont compliquées de sténose distale de la veine. Le diagnostic a été suspecté devant la diminution du débit pendant la séance de dialyse, l'existence d'une hyperpression dans la circulation extracorporelle, la présence de douleur et l'aspect tendu de la veine qui ne se collabe pas à la surélévation du membre supérieur. Le diagnostic a été confirmé par la réalisation d'un écho-doppler artériel et veineux du membre supérieur concerné chez deux patients complété par une fistulographie chez les quatre patients. Les anévrismes: on a enregistré trois faux-anévrysmes et un seul anévrysme vrai soit 12,90% de l'ensemble des complications. Le diagnostic était purement clinique par la constatation d'une tuméfaction en regard de la voie d'abord (anastomotique) ou légèrement à distance (juxta-anastomotique). Devant la suspicion d'une sténose sur le trajet de la FAV, une fistulographie a été réalisée chez un patient. Les complications ischémiques: Trois FAV (9,67%) se sont compliquées d'un syndrome de vol avec ischémie du membre supérieur et abolition des pouls radial et cubital. L'infection dans 3 cas (9,67%): Une FAV radio-céphalique s'est compliquée d'une nécrose cutanée avec infection; Une FAV huméro-céphalique s'est compliquée d'une infection avec hémorragie sévère; Le troisième cas était une infection de prothèse huméro-axillaire (PTFE). L'hémorragie: Deux FAV huméro-céphaliques (6,45%) se sont compliquées d'hématome en regard de l'abord chirurgical et ont cédé par simple compression et surélévation du membre. L'hyperdébit: une FAV (3,22%) s'est compliquée d'un hyperdébit sans retentissement sur le lit d'aval avec à l'examen clinique une circulation veineuse collatérale importante; il s'agissait d'une fistule huméro-céphalique.

**Tableau 1:** Complications survenues au cours de notre étude

Complications	Nombre de cas	Pourcentage par rapports à l'ensemble des complications
Thrombose précoce (avant ou début dialyse)	6	19,35%
Thrombose tardive (après dialyse)	8	25,80%
Sténose	4	12,90%
Anévrisme	4	12,90%
Infection	3	9,67%
Complications ischémiques	3	9,67%
Complications hémorragiques	2	6,45%
Hyperdébit	1	3,22%

## Résultats

**Figure 1:**
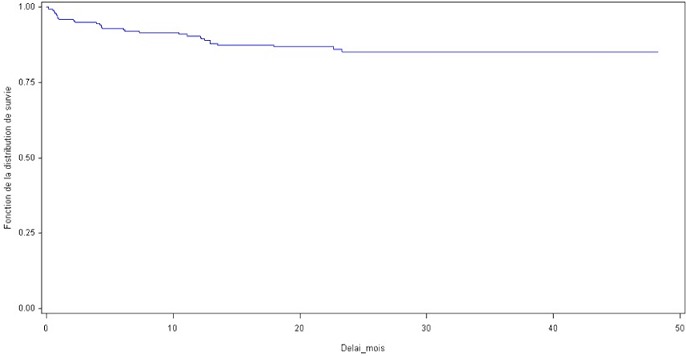
Courbe de perméabilité primaire des FAV durant les années d’étude avec un taux globale de réussite de 83%

**Figure 2:**
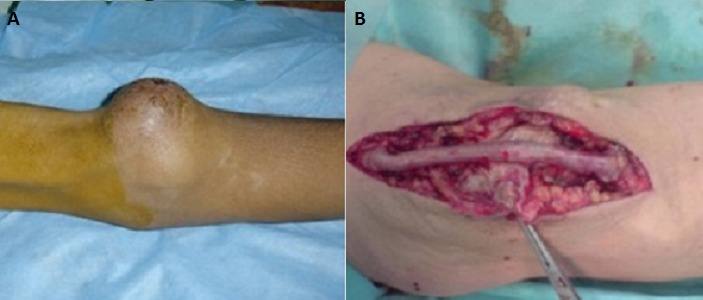
A) Faux anévrysme juxta-anastomotique sur FAV huméro-céphalique; B) greffon veineux en veine fémorale superficielle après mise à plat du faux anévrisme

**Figure 3:**
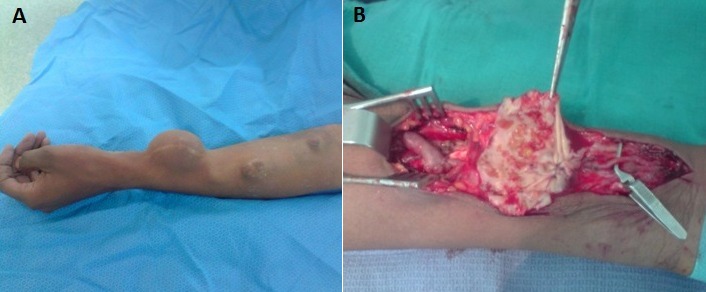
A) faux anévrysme anastomotique sur FAV radio-céphalique; B) mise à plat + réimplantation de la veine dans l'artère radiale

**Figure 4:**
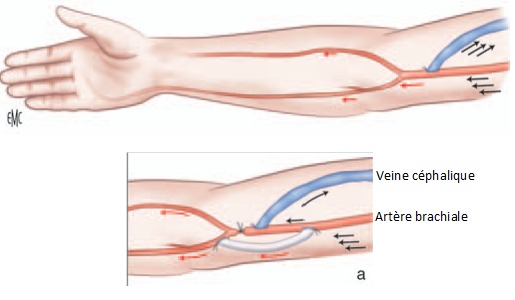
Représentation schématique de l'intervention de DRIL

**Figure 5:**
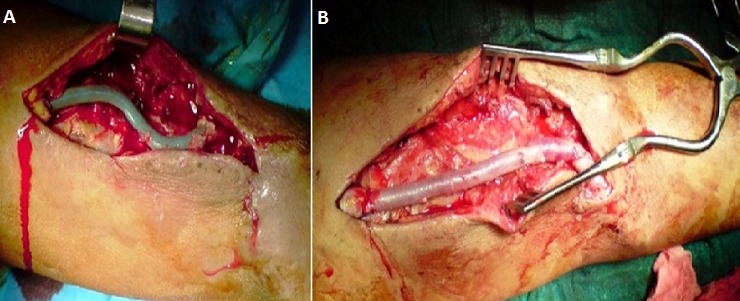
A) infection d'un PAV huméro-axillaire pour hémodialyse; B) traitement chirurgical par ablation de la prothèse infectée + greffon veineux

Pendant la durée d’étude on a eu 31 complications parmi les 200 fistules réalisées, soit 14,9%; avec un taux global de réussite de 83% (Figure 1). Les thromboses ont été traitées chirurgicalement par réimplantation ou par création d'une nouvelle FAV selon l'indication; Pour les 6 thromboses précoces: on a réussi à conserver 2 FAV par réimplantation de la veine en question dans l'artère correspondante puisque la veine était bien développée dans les deux cas. Alors que dans les 6 cas restants on a confectionné une nouvelle FAV. Pour les 8 thromboses tardives: on a conservé 6 FAV par réimplantation de la veine sur l'artère correspondante, alors que dans les 2 autres cas, une nouvelle FAV a été confectionnée. Trois sténoses ont bénéficié d'une angioplastie transluminale (ATL) par voie endovasculaire et une a été traitée par réimplantation de la veine céphalique de l'avant bras sur l'artère radiale. Deux faux anévrysmes sur FAV huméro-céphaliques ont été traité par mise à plat avec fermeture de l'orifice au niveau de la veine; un anévrysme vrai sur FAV huméro-céphalique a été traité par mise à plat avec greffon veineux (Figure 2); un faux anévrysme sur FAV radio-céphalique a été traité par mise à plat avec réimplantation (Figure 3); Les FAV compliquées d'ischémie ont subit un traitement chirurgical qui a consisté en l'intervention de DRIL (Revascularisation Distale avec Ligature Intermédiaire). Le principe de cette intervention a consisté en une ligature de l'artère en aval de la FAV, visant à supprimer le phénomène de vol, et l'interposition d'un pontage entre l'artère, en amont de la FAV et immédiatement en aval de celle-ci, visant à rétablir une perfusion distale satisfaisante (Figure 4). Deux complications infectieuses ont été traitées par antibiothérapie adapté au prélèvement fait lors du parage; une infection de prothèse huméro-axillaire (PTFE) (Gortex) a été traité chirurgicalement par ablation de la prothèse avec interposition d'un greffon veineux en veine fémorale superficielle (Figure 5). Une fistule huméro-céphalique s'est compliquée d'un hyperdébit. Le traitement a consisté en la supression de la FAV et la création d'une autre huméro-céphalique au niveau du membre supérieur controlatéral. On a regretté dans notre série un seul cas de décès. Ainsi on a pu conserver 22 FAV soit 70,96%; on a confectionné une nouvelle FAV dans 8 cas soit 25,80% et on a adressé une patiente (3,22%) pour pose d'un cathéter veineux tunnelisé permanent.

## Discussion

Les complications tardives sont les plus fréquentes. Le plus souvent il s'agit de sténose sur le versant veineux des FAV ou de thromboses. Les anévrysmes surviennent généralement sur les fistules anciennes et correspondent aux traumatismes répétés des points de ponction. Les complications infectieuses sont plus rares mais plus graves.

**Sténoses et thromboses**: La sténose se constitue lentement et peut être suspectée devant la survenue d'un débit insuffisant en dialyse ou de difficultés de ponction [2]. Une sténose péri-anastomotique sur FAV proximale peut être traitée par ATL. Elle peut également être traitée par chirurgie, avec confection d'une nouvelle anastomose plus proximale. La plupart des auteurs ont considèré la chirurgie comme le traitement de référence pour cette complication [3, 4]. L’évolution naturelle des sténoses est la thrombose. Elle doit être prévenue par une surveillance régulière de la voie d'abord au cours des dialyses. Le traitement utilise principalement les techniques endovasculaires en première intention [3].

**Anévrysmes:** Dan l’étude de Bakran A et Mickley concernant les anévrismes sur FAV, ils se sont réfèré à des segments de veine dilatée, dont le diamètre dépasse 1,5 à 2 fois le diamètre de la veine adjacente [5]. Plusieurs types d'anévrysmes peuvent se développer, soit sur le versant artériel, anastomotique, soit le long de la voie de drainage. Certains sont des anévrysmes vrais artériels ou veineux, d'autres sont des faux anévrysmes. Les fréquences des anévrysmes sur FAV rapportées dans la littérature ont été de 0% à 17% pour les FAV natives et de 0% à 7% pour les pontages par prothèse en Polytetrafluoroethylène (PTFE) [6]. Le traitement est chirurgical, et le geste à faire dépend surtout du siège de l'anévrysme par rapport à la FAV allant de la simple mise à plat avec fermeture de l'orifice d'alimentation, à la mise à plat avec greffon [7]. Dans notre série, on a enregistré trois faux-anévrysmes et un seul anévrysme vrai soit 12,90% de l'ensemble des complications et 2% de l'ensemble des FAV.

**Complications ischémiques ou hémodétournements**: Elles sont redoutables et s'observent particulièrement en cas de fistule proximale surtout chez les patients diabétiques et âgés. Le risque d'hémodétournement varie, en fonction du type de FAV, de 1% à 2% pour les FAV situées au niveau de l'avant bras, jusqu’à 5% à 15% pour les fistules situées au niveau du coude et du bras [8]; et de 3 à 4% pour les PAV [9]. Les ischémies de la main sont le plus souvent d'origine artérielle [8, 9] et plus rarement veineuse. Il s'agit d'un « phénomène de vol » retrouvé dans 80% des cas. Wixon et coll [10] ont montré que la survenue de complications ischémiques faisait intervenir plusieurs parametres tels que le débit, les pressions au niveau et en aval de la fistule, l’état du lit vasculaire périphérique et la présence des collatérales situées de part et d'autre de la fistule. Elle peut être isolée ou associée à un hyperdébit et le traitement dépend de la sévérité de l'ischémie et/ou de l'hyperdébit [11]. Dans notre série on a réalisé la technique de DRIL [12] pour traiter les complications ischémiques. L’évolution était favorable avec amélioration complète des symptômes de l'ischémie ce qui rejoint la littérature (Tableau 2).

**Tableau 2:** Résultats de l'intervention de DRIL selon les différentes séries

Auteurs	Année de publication	Nombre d'intervenion type DRIL réalisées	Taux de succès%	Taux de peméabilité de la FAV%
Scjanzer et coll.	1992	14	93	82
Haimov et coll.	1996	23	96	73
Katz et coll.	1996	6	83	100
Berman et coll.	1997	21	100	94
Lazarides et coll.	1998	7	94	-
Stierli et coll.	1998	6	100	100
Knox et coll.	2002	52	90	83
Notre série	2011	3	100	100

**Hyperdébi** : L'accroissement du débit est conditionné par l'artère donneuse et sa capacité à se dilater, mais aussi par l'ancienneté de l'accès. C'est une complication grave mais rare des FAV; elle se voit chez 1% à 8% de patients avec des signes cliniques graves. Les pontages artério-veineux qui développent rapidement une sténose de l'anastomose veineuse sont moins pourvoyeurs d'hyperdébit. La réduction d'un hyperdébit s'impose s'il est mal toléré sur le plan cardiaque, ou devant l'apparition d'une ischémie distale par vol vasculaire [13]. Les techniques proposées pour la réduction de débit sont nombreuses et de complexité variable [13]. La technique de DRIL est exclusivement utilisée pour le traitement du syndrome de vol, alors que la technique de Banding chirurgicale peut être utilisée pour le traitement du syndrome de vol et de l'insuffisance cardiaque causés par l'hyperdébit [13]. Dans notre série une FAV s'est compliquée d'hyperdébit sans retentissement sur le lit d'aval soit 3,22% de l'ensemble des complications et 0,5% de l'ensemble des FAV.

**Infection**: Elles ont fait le plus souvent l'objet dans un premier temps d'un traitement conservateur associant au drainage de la plaie et aux soins locaux une antibiothérapie adaptée. En cas d'un PAV par prothèse en PTFE, et en l'absence d'amélioration rapide des signes locaux, l'ablation de la prothèse manifestement infectée doit être rapidement effectuée et reste le traitement le plus sure et le plus efficace. Le taux d'infection sur FAV natives est de 2 à 3%, et sur greffon prothétique varie de 11% à 35%. Les infections secondaires aux ponctions peuvent être traitées par une résection segmentaire du greffon avec pontage [14–16]. Dans notre série trois FAV se sont compliquées d'une infection, dont une est une infection de prothèse en PTFE.

**Complications hémorragiques**: Leurs causes sont variées. Il peut s'agir d'un défaut d'hémostase, notamment chez les malades sous traitement antiagrégant. Il peut s'agir d'un saignement en rapport avec de fines veinules artérialisées. Ce peut être enfin, un traumatisme lors de la tunnellisation d'un pontage. Les hémorragies lorsqu'elles sont extériorisées ne peuvent être traitées par compression locale et imposent la reprise chirurgicale. Il en est de même pour l'hématome postopératoire, qui peut également retarder l'incorporation d'une prothèse et son utilisation. Dans les deux cas, l'hématome augmente le risque infectieux [1]. Dans notre série deux FAV huméro-céphaliques se sont compliquées d'hématome en regard de l'abord chirurgical ayant cédé par simple compression et surélévation du membre.

## Conclusion

Les complications des FAV pour hémodialyse chronique constituent la principale cause de morbidité chez 1'hémodialysé chronique, c'est pourquoi il est primordial de s'appliquer lors de leur création, et d'accorder le maximum d'attention lors de leur manipulation. Ceci implique le chirurgien, le néphrologue, les infirmiers et le patient lui-même, ainsi que la mise en place d'un programme de surveillance des FAV. Les techniques d'angioplastie endoluminale offrent les meilleures perspectives dans la gestion des complications.
